# Iowa Medical Society

**Published:** 1855-07

**Authors:** 


### IOWA MEDICAL SOCIETY.

The Iowa Medical Society met in this city on Thursday, June 14th. There were present of old members, about twenty-five. A number of new members were admitted. The proceedings will be published in the next number of this Journal.

The meeting being small caused, perhaps, more familiarity of intercourse, and the proceedings assumed rather a colloquial turn, which in our opinion should ever characterize such associations.

It cannot be denied that in the attempt to systematize and give a more formal and scientific aspect to our medical associations, we have to some extent limited the practical results, which we should expect to derive from the professional intercourse secured by them. The usual mode of appointing committees with special duties and subjects of report, is well enough as tending to give permanency and method to our transactions, but so far as they prevent a familiar and free expression by individual members, upon any and all subjects of medical interest, they mar the real good and thwart what should be the real design of such periodical assemblages of medical men. Formality, beyond what is required to systematize, always obtains at the expense of the vitality of effort.

It is too commonly the case, that a failure to report on the part of committees on given subjects, leaves them untouched by the Society, and even where reports are prepared, an abstract is all we get, and they pass into the archives, unread and without discussion.—We know it is said, we have not the time to do otherwise. But we should take time. It strikes us that one of the highest objects of these associations should be. and is, to elicit a free, open, familiar and mutual exposition of the theories, observations, and practical bearings of each and every member, upon each and every subject of interest which may arise.

Many valuable facts which are known to, and acted upon, by many of our country friends, remote from media of publication, which they hence modestly consider unworthy the trouble of placing in print, would, by this unrestrained intercourse be thrown out, and many a hint would be elicited which for the development, might render of high importance to the profession. The collision of minds, always a pleasant excitement, would arouse in members, a desire for partaking in discussions. Extemporaneous speaking cannot but be improving to our younger brethren.

We think no one who attended the late meeting in this city, failed to see and feel the effects to which we have alluded. Neither the President nor Secretary of the previous meeting being present, and no records being in our hands, we were from necessity, a little unsystematic and less governed by rules or restrained by strict conformity, and a wide scope was given to every member to make any kind of communication and in his own way, than is usual.

It is to be regretted that a larger number of our brethren do not attend the meetings of the society. We know it is at the sacrifice of time and money, and often, even, of the interests of patients; but no medical man should neglect wilfully, nay, even at some loss, any opportunity of advancing the interest of his profession, by a free intercourse with others, and an open declaration of what he may believe of general value, and a candid and fair investigation and trial of what may be suggested by others. Besides, these meetings tend strongly to fraternize the profession; to unite their efforts, and by a union of purpose and action to sustain the dignity of the science against fraud and quackery. When we, form the constant and general habit of thus meeting and cultivating good feelings as well as true science, we shall no longer have doctors called *natural enemies;* and pretenders must seek some other basis than our differences to build their own claims upon.

We were much pleased to see the society unanimously recommending this Journal as a suitable medium of communication on medical subjects, and even a strong inclination expressed by some, to pledge themselves to sustain it as a monthly publication instead of a bi-monthly issue, as it now is.

They were also pleased to sanction unanimously, the course of the Medical department of the University, and to commend it to the fostering care of the State and the liberal patronage of the profession at large. The next meeting was appointed at Ottumwa, Wapello county, where we hope to meet a large number of our brethren on the second Wednesday in June, 1856.

